# Profiling of gene expression in the brain associated with anxiety-related behaviors in the chronic phase following cranial irradiation

**DOI:** 10.1038/s41598-022-17310-z

**Published:** 2022-08-01

**Authors:** Hae-June Lee, Joong-Sun Kim, Changjong Moon, Yeonghoon Son

**Affiliations:** 1 Division of Radiation Biomedical Research, Korea Institute of Radiological & Medical Sciences (KIRAMS), Seoul, 01812 Republic of Korea; 2 Department of Veterinary Anatomy and Animal Behavior, College of Veterinary Medicine and BK21 FOUR Program, Chonnam National University, Gwangju, 61186 Republic of Korea

**Keywords:** Neuroscience, Cancer

## Abstract

Although the brain is exposed to cranial irradiation in many clinical contexts, including malignant brain tumor therapy, such exposure can cause delayed neuropsychiatric disorders in the chronic phase. However, how specific molecular mechanisms are associated with irradiation-induced behavioral dysfunction, especially anxiety-like behaviors, is unclear. In the present study, we evaluated anxiety-like behaviors in adult C57BL/6 mice using the open-field (OF) and elevated plus maze (EPM) tests 3 months following single cranial irradiation (10 Gy). Additionally, by using RNA sequencing (RNA-seq), we analyzed gene expression profiles in the cortex and hippocampus of the adult brain to demonstrate the molecular mechanisms of radiation-induced brain dysfunction. In the OF and EPM tests, mice treated with radiation exhibited increased anxiety-like behaviors in the chronic phase. Gene expression analysis by RNA-seq revealed 89 and 106 differentially expressed genes in the cortex and hippocampus, respectively, following cranial irradiation. Subsequently, ClueGO and STRING analyses clustered these genes in pathways related to protein kinase activity, circadian behavior, and cell differentiation. Based on our expression analysis, we suggest that behavioral dysfunction following cranial irradiation is associated with altered expression of *Cdkn1a*, *Ciart*, *Fos*, *Hspa5*, *Hspb1* and *Klf10*. These novel findings may provide potential genetic targets to investigate for the development of radioprotective agents.

## Introduction

Radiation therapy is commonly used to treat primary and secondary brain tumors, including glioma and glioblastoma^1,2^. However, exposure to ionizing radiation is associated with several side effects, including anxiety, depression, and cognitive impairment. The delayed effect of radiation-induced cognitive dysfunction has been reported in patients (> 6 months post-irradiation), indicating that these progressive memory deficits remain a significant risk^3^. A previous clinical study also reported that 30% and 17% of patients with a brain tumor suffer from anxious mood and depressed mood, respectively^4^. In addition, a previous longitudinal study analyzed anxiety symptoms in patients with a primary brain tumor receiving radiation therapy^5^, which might have a detrimental impact on the quality of life of cancer survivors.

Several preclinical studies have detected radiation-induced cognitive dysfunction in animal models using behavioral tests. For example, irradiated mice exhibited an adverse change in cognitive function during a contextual fear conditioning task or the Barnes maze test^6,7^. In addition, cranial radiation was found to lead to abnormal hippocampal-dependent cognitive function at 1 or 3 months after irradiation in the novel object recognition memory task^8,9^. Moreover, depression-like behavior was reported in adult mice at 90 days following cranial irradiation, indicating that ionizing radiation might progressively affect emotional function^10^. Increased anxiety-like behavior has been reported in a variety of rodent models, including models of obesity^11^, chronic stress^12^, and social disruption^13^. In addition, mice treated with radiation and temozolomide were shown to exhibit increased anxiety-like behavior at 6 weeks following cranial irradiation^14^. However, few behavioral studies on anxiety-like behavior in the chronic phase following cranial irradiation have been published to date.

To demonstrate how cranial irradiation affects brain functions, we investigated anxiety-like behavior in C57BL/6 mice at 3 months after cranial irradiation. In addition, we investigated the gene expression profiles in the cortex and hippocampus of adult mice after cranial irradiation to determine the molecular mechanisms by which ionizing radiation affects brain function and to identify novel candidate genes associated with behavioral dysfunction.

## Results

### Anxiety-like behavior in C57BL/6 mice in the chronic phase following cranial irradiation

**Figure 1:**
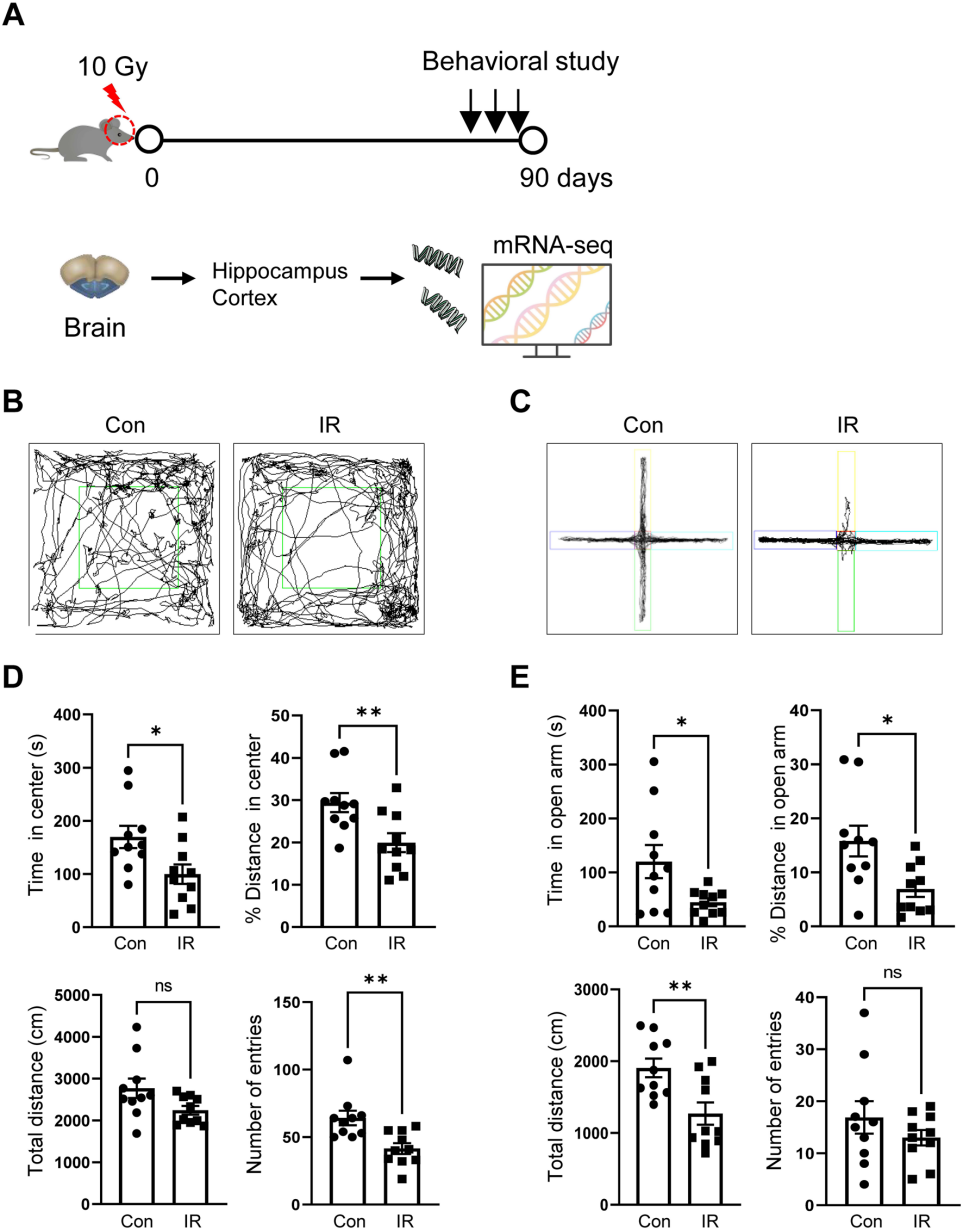
Anxiety-related behavior during the chronic phase following cranial irradiation. (**A**) Schematic diagram of our experimental procedure. (**B**) Representative tracking of mouse movement during the open-field (OF) test. (**C**) Representative tracking of mouse movement during the elevated plus maze (EPM) test. (**D**) The time spent in the center, % distance traveled from the center, total distance traveled, and number of entries in the OF apparatus were assessed in sham-irradiated (Con) and 10 Gy-irradiated (IR) mice. (**E**) The time spent in the open arm, % distance traveled in the open arm, total distance traveled, and number of entries in the EPM test were assessed in the Con and IR groups. Data are expressed as the mean ± SE (*n* = 10 per group). *p < 0.05 and **p < 0.01 *vs.* the Con group.

A schematic diagram of this study is shown in Fig. 1A. To investigate whether cranial irradiation alters anxiety-related behaviors in the chronic phase, we first observed the behavior of mice in an OF apparatus. Irradiated mice spent significantly less time in the center, traveled less distance, and made fewer entries into the center of the apparatus, which are indications of enhanced anxiety-like behavior (Fig. 1B,C). However, no difference in total distance traveled was evident between controls and irradiated mice at 3 months post-irradiation. A similar pattern of behavior was observed in the EPM test. Mice irradiated with 10 Gy spent significantly less time and traveled less distance in the open arms of the EPM (Fig. 1D,E). In addition, irradiated mice traveled a significantly lower distance in the EPM, indicating a reduction in locomotor activity under anxiogenic conditions (Fig. 1E). These findings indicate that cranial irradiation enhanced anxiety-like behavior in the chronic phase following cranial irradiation.

### Differentially expressed genes (DEGs) in the cortex and hippocampus after cranial irradiation

**Figure 2:**
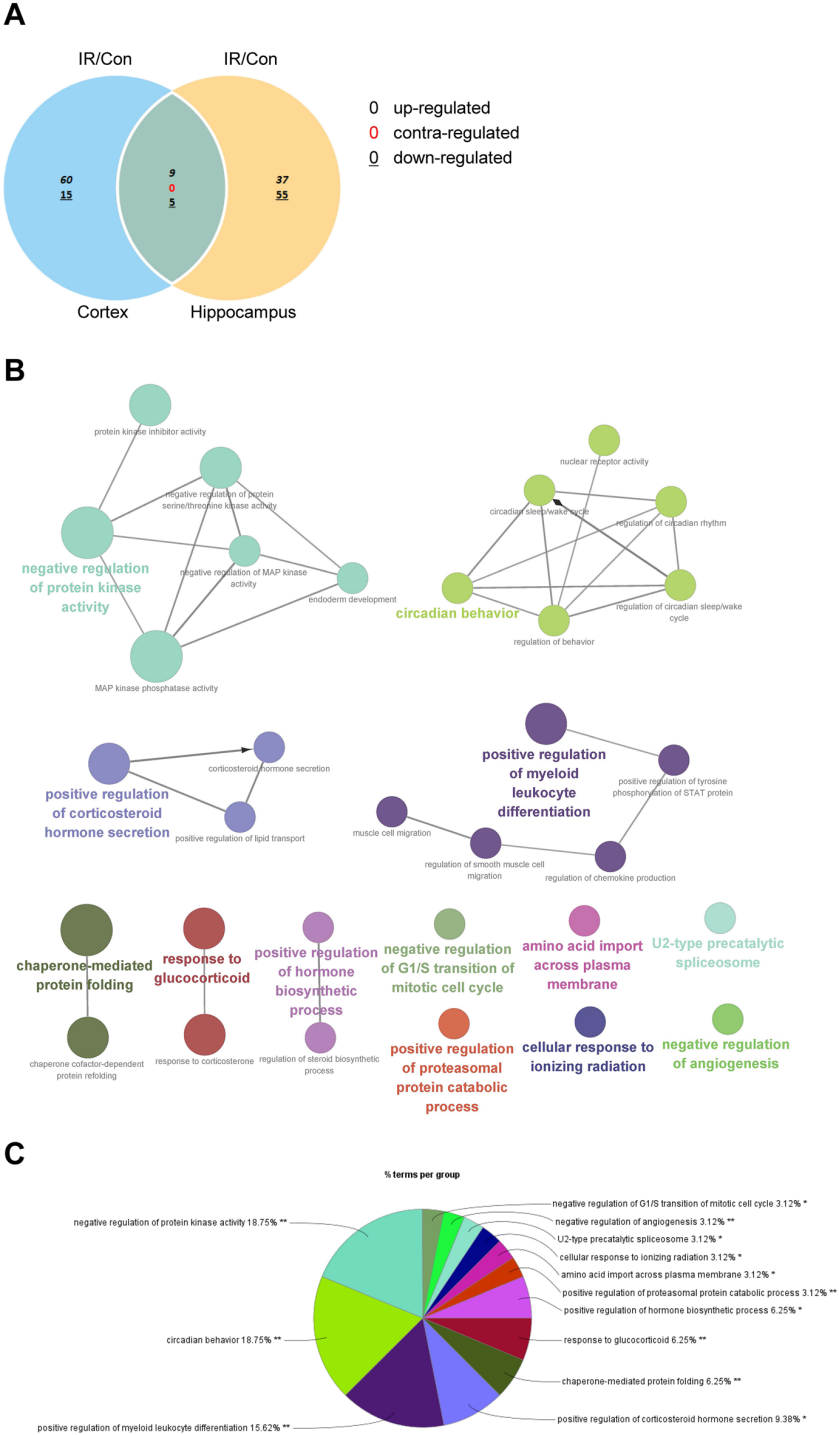
Gene ontology (GO) analysis of RNA-seq screening data. (**A**) Venn diagram of differentially expressed genes (DEGs) in the cortex and hippocampus between sham-irradiated (Con) and 10 Gy-irradiated (IR) mice. (**B**,**C**) ClueGO network analysis of enriched categories in the cortex and/or hippocampus after cranial irradiation. GO terms describing molecular interactions among targets are represented as nodes, and node size represents the term's enrichment significance.

To determine the radiation-specific gene profiles related to anxiety-like behavior, total RNA-seq was carried out in the cortex and hippocampus at 3 months after cranial irradiation. As a criterion for the RNA-seq data, we assumed a fold change of 1.5 and a *p*-value of 0.05. Compared to their expression in the control group, 89 and 106 genes were differentially expressed in the cortex and hippocampus, respectively, of the irradiated mice (Fig. 2A). In detail, 75 and 92 genes were uniquely dysregulated in the cortex and hippocampus, respectively, while 97 genes were upregulated and 70 genes were downregulated. Moreover, 14 genes were dysregulated in both regions, while 9 genes were upregulated and 5 genes were downregulated.

### Functional gene annotation and enrichment analyses

The clustering heatmap of the 181 DEGs in the cortex and/or hippocampus at 3 months following cranial irradiation is shown in Supplementary Fig. 1. The 181 DEGs modified by cranial irradiation in the functional network were clustered by ClueGO into functional groups related to Gene Ontology (GO) biological processes, resulting in 13 terms (Table 1). The highest number of identified genes were classified directly under negative regulation of protein kinase activity (GO:0006469, GO:0071901), followed by regulation of circadian rhythm (GO:0042752) and muscle cell migration (GO:0014812). The genes whose expression was induced by radiation were mainly associated with protein kinase activity (18.75%), circadian behavior (18.75%), and myeloid leukocyte differentiation (15.62%) (Fig. 2B,C). Other biological processes, such as corticosteroid hormone secretion, chaperone-mediated protein folding, and the response to glucocorticoids, were also represented (Fig. 2C).

**Table 1:** Gene Ontology (GO) terms of genes enriched by CuleGO analysis.

Process	Term ID	Term name	No. of Genes	Associated genes found
Cell cycle	GO:2000134	Negative regulation of G1/S transition of mitotic cell cycle	3	Cdkn1a, Klf4, Plk3
Angiogenesis	GO:0016525	Negative regulation of angiogenesis	6	Cd59a, Gadd45a, Gm20498, Klf2, Klf4, Thbs4
Spliceosome	GO:0071005	U2-type precatalytic Spliceosome	3	Cwc27, Lsm5, Lsm8
Response to radiation	GO:0071479	Cellular response to Ionizing radiation	4	Cdkn1a, Egr1, Gadd45a, Hspa5
Transport	GO:0089718	Amino acid import Across plasma membrane	3	Ntsr1, Per2, Slc6a13
Metabolic process	GO:1901800	Positive regulation of proteasomal protein catabolic process	6	Hspa1b, Klhl40, Nupr1, Plk3, Trib1, Trib2
Hormone metabolic process	GO:0046886 GO:0050810	Positive regulation of hormone biosynthetic process regulation of steroid biosynthetic process	3 4	Bmp6, Egr1, Gh Bmp6, Egr1, Gh, Nr1d1
Response to steroid hormone	GO:0051384 GO:0051412	Response to glucocorticoid Response to corticosterone	8 4	Bmp6, Cdkn1a, Crh, Dusp1, Fos, Fosb, Ptgds, Sstr2 Cdkn1a, Crh, Fos, Fosb
Protein folding	GO:0061077 GO:0051085	Chaperone-mediated protein folding Chaperone cofactor-Dependent protein refolding	6 4	Dnajb5, Hspa1b, Hspa5, Hspb1, Pdia4, Sdf2l1 Dnajb5, Hspa1b, Hspa5, Sdf2l1
Steroid hormone secretion	GO:0032370 GO:0035930 GO:2000848	Positive regulation of Lipid transport Corticosteroid hormone secretion Positive regulation of corticosteroid hormone secretion	4 3 3	Bmp6, Crh, Ecrg4, Ntsr1 Bmp6, Crh, Ecrg4 Bmp6, Crh, Ecrg4
Cell differentiation	GO:0014812 GO:0032642 GO:0014910 GO:0002763 GO:0042531	Muscle cell migration regulation of Chemokine production Regulation of smooth muscle cell migration Positive regulation of myeloid leukocyte differentiation Positive regulation of tyrosine phosphorylation of STAT protein	6 4 4 5 3	Ccl5, Ccn3, Egr1, Nr4a3, Thbs4, Trib1 Ccl5, Csf1r, Egr1, Klf4 Ccl5, Egr1, Nr4a3, Trib1 Ccl5, Csf1r, Fos, Klf10, Trib1 Ccl5, Csf1r, Gh
Behavior	GO:0042752 GO:0050795 GO:0048512 GO:0042745 GO:0042749 GO:0004879	Regulation of circadian rhythm Regulation of behavior Circadian behavior Circadian sleep/wake cycle Regulation of circadian Sleep/wake cycle Nuclear receptor activity	6 5 5 3 3 3	Cort, Klf10, Nr1d1, Per2, Ptgds, Sik1 Cort, Crh, Nr1d1, Nr4a3, Ptgds Ciart, Cort, Egr1, Nr1d1, Ptgds Cort, Nr1d1, Ptgds Cort, Nr1d1, Ptgds Nr1d1, Nr4a1, Nr4a3
Kinase activity	GO:0033549 GO:0007492 GO:0004860 GO:0006469 GO:0043407 GO:0071901	MAP kinase Phosphatase activity Endoderm development Protein kinase inhibitor activity Negative regulation of Protein kinase activity Negative regulation of MAP kinase activity Negative regulation of Protein serine/threonine kinase activity	4 4 5 11 5 7	Dusp1, Dusp4, Dusp5, Dusp6 Arc, Dusp1, Dusp4, Dusp5 Cdkn1a, Hspb1, Spry4, Trib1, Trib2 Cdkn1a, Dusp1, Dusp4, Dusp5, Dusp6, Gadd45a, Hspb1, Shb, Spry4, Trib1, Trib2 Dusp1, Dusp4, Dusp5, Dusp6, Spry4 Cdkn1a, Dusp1, Dusp4, Dusp5, Dusp6, Gadd45a, Spry4

**Figure 3:**
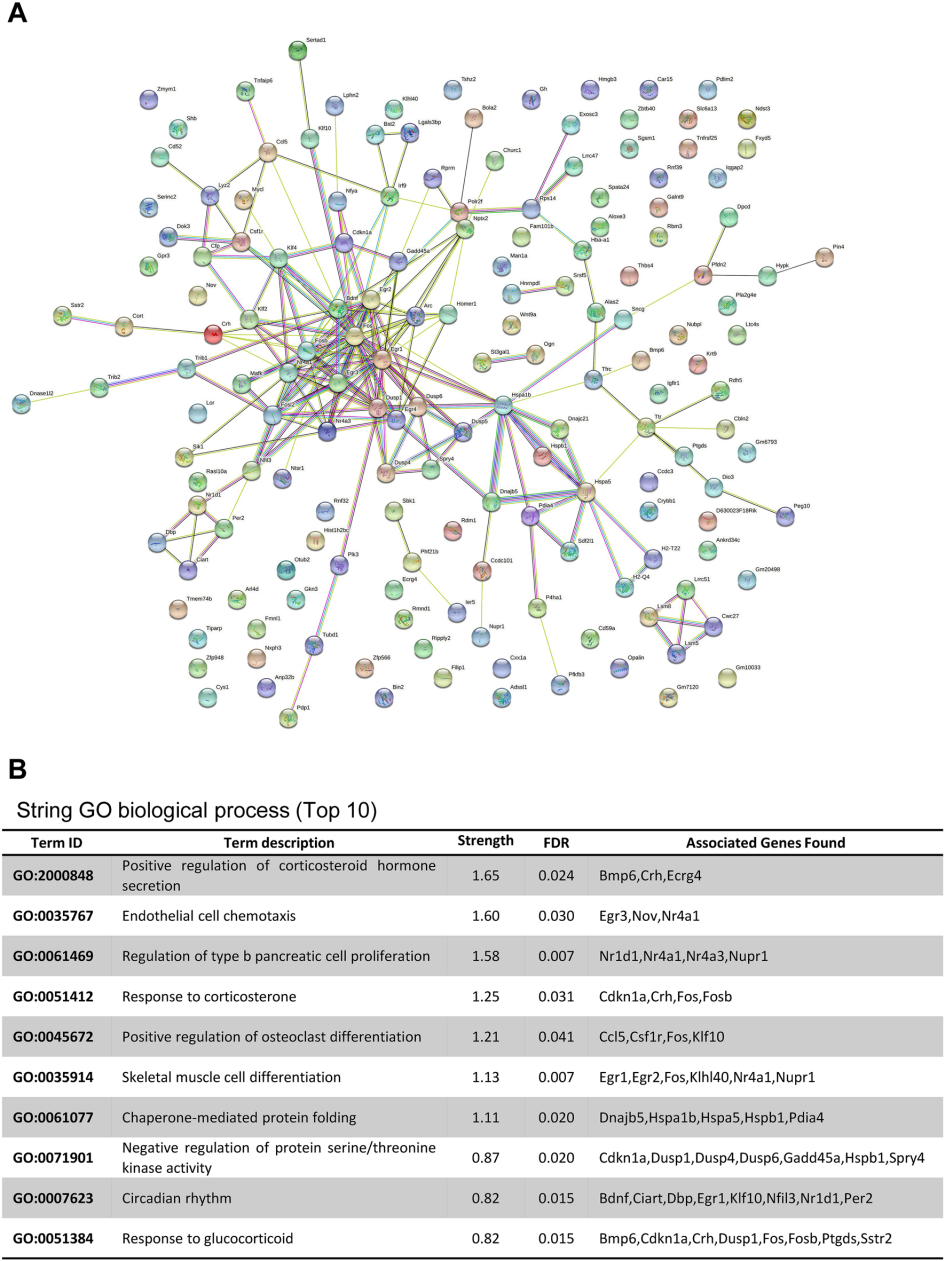
STRING network and enrichment analyses. (**A**) STRING analysis of 181 differentially expressed genes (DEGs) obtained upon RNA-seq analysis of the cortex and hippocampus between sham-irradiated (Con) and 10 Gy-irradiated (IR) mice. (**B**) The top 10 Gene Ontology (GO) biological processes that were functionally enriched in genes in the network were selected in order of highest strength with an FDR < 0.05.

To further demonstrate the perturbation induced by cranial irradiation, we examined targets of the DEGs using STRING analysis, selecting the top 10 GO terms (Fig. 3A,B). Among these, the most interesting identified GO terms were “circadian behavior”, “cell differentiation”, and “protein kinase activity”. In the network identified with the STRING analysis, whereas 8 and 6 genes were associated with circadian rhythm (GO:0007623) and skeletal muscle cell differentiation (GO:0035914), another 7 genes displayed association with protein kinase activity (GO:0071901).

### qRT–PCR validation

**Figure 4:**
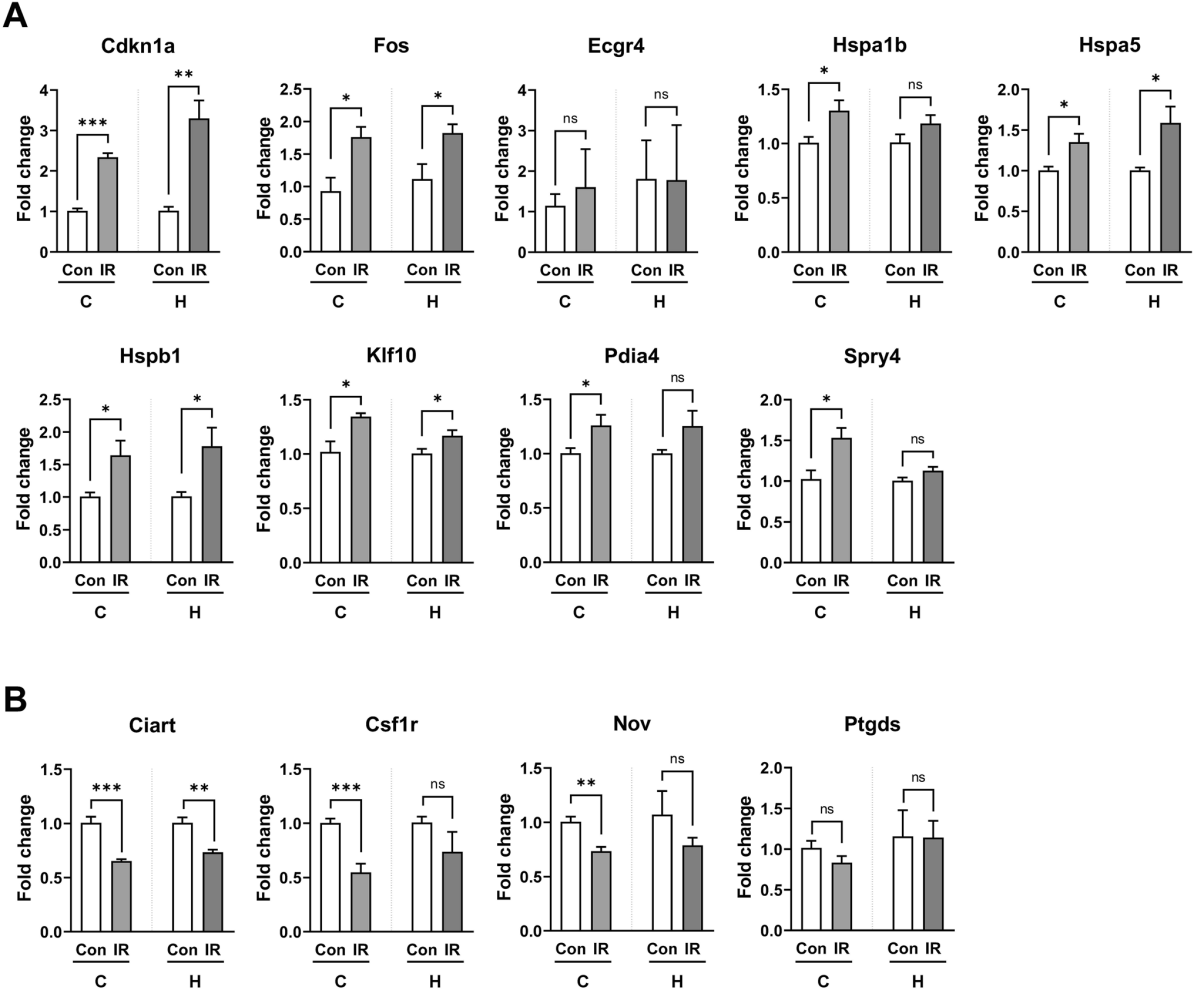
Changes in 13 selected differentially expressed genes (DEGs) in the cortex and hippocampus of mouse brains after cranial irradiation. The bar graphs show the relative expression levels of upregulated (**A**) and downregulated (**B**) genes from RNA-seq data collected from both the cortex and hippocampus. Data are expressed as the mean ± SE (*n* = 5 per group). *p < 0.05, **p < 0.01, and ***p < 0.001 *vs.* the Con group.

Since many DEGs were associated with several biological processes of the brain, the obtained RNA-seq data were validated by qRT–PCR. Notably, 30 genes clustered by both ClueGO (47 genes) and STRING (35 genes) analyses at 3 months post-irradiation were selected (Table 2). To validate the RNA-seq data demonstrated the genes that were significantly up- or downregulated by cranial irradiation in both the cortex and hippocampal tissue, we examined the gene expression levels by qRT–PCR analysis. There were 9 DEGs whose levels were significantly upregulated by radiation exposure in both the cortex and hippocampus of the mouse brain. In addition, 4 genes were significantly downregulated in the cortex and hippocampal tissue. qRT–PCR analysis confirmed that 8 and 3 genes were significantly upregulated (Fig. 4A) and downregulated (Fig. 4B), respectively, in the chronic phase following cranial irradiation. Among them, the expression patterns of 6 genes, *Cdkn1a*, *Ciart*, *Fos*, *Hspa5*, *Hspb1* and *Klf10*, were identical in both the cortex and hippocampus, suggesting specific and chronic changes in the expression of these genes after radiation exposure. In contrast, other genes whose levels were identified as changed after cranial irradiation did not follow the same expression pattern.

**Table 2:** Genes selected for validation by qRT-PCR.

Gene symbol	Fold change (Cortex)	Fold change (Hippocampus)	NCBI sequence	Primer pair	Length (bp)
Bmp6	0.814	0.639**	NM_007556.3	F: CAGACTACAACGGCAGTGAG R: CCTTTGGGTGCAATGATCCA	103
Ccl5	3.974	0.035*	NM_013653.3	F: TGCCCACGTCAAGGAGTATT R: ACTTCTTCTCTGGGTTGGCA	107
Cdkn1a	2.460***	2.913***	NM_007669.5	F: AAGTGTGCCGTTGTCTCTTC R: CGAAGTCAAAGTTCCACCGT	114
Ciart	0.517***	0.647**	NM_001033302.2	F: GCCACAGTTTGCCAGTAACA R: GGCTCTGGGTGTCCTTTAGT	103
Crh	1.555**	1.037	NM_205769.3	F: CCGCAGCCCTTGAATTTCTT R: AGCGGGACTTCTGTTGAGAT	114
Csf1r	0.583***	0.537***	NM_001037859.2	F: CCTACCGTTGTACCGAGCTT R: CTCCTGTGCCAGCAAATTCC	101
Dnajb5	1.602***	1.117	NM_001355438.1	F: TGAGTGACCCTAAGAAGCGG R: AAGGAAGCAAAGGTGGCATG	139
Dusp1	1.529***	1.124	NM_013642.3	F: TCTCCCCGAACTTCAGCTTC R: CTGTGGTAGTAGAGGTGCCC	129
Dusp4	1.742***	0.991	NM_176933.4	F: GCTCTAAAACCAAGGCCCTG R: CGAGGTAGAGGAAAGGGAGG	144
Dusp6	1.777***	1.150	NM_026268.3	F: AACCTGTCCATGAACGATGC R: TCCTTTCGAAGTCAAGCAGC	100
Ecrg4	4.054***	1.351*	NM_024283.3	F: GCAGTTCCTCTACATGGGCT R: CCAATGGCCGCATCTTCATC	135
Egr1	2.106***	1.104	NM_007913.5	F: ATGAGAAGGCGATGGTGGAG R: CTCACGAGGCCACTGACTAG	154
Fos	2.348***	1.730***	NM_010234.2	F: GGGCTGCACTACTTACACGT R: TGCCTTGCCTTCTCTGACTG	169
Fosb	1.923***	0.990	NM_008036.2	F: AACCAGCTACTCAACCCCAG R: CTTCTCGGGGTCTTCTAGGC	140
Gadd45a	1.566**	1.078	NM_007836.1	F: CATTTCACCCTCATCCGTGC R: TCGTTCTCCAGTAGCAGCAG	101
Hspa1b	1.715***	1.324**	NM_010478.2	F: GGACATCAGCCAGAACAAGC R: TGTGTAGAAGTCGATGCCCT	133
Hspa5	1.582***	1.512***	NM_001163434.1	F: TCCTTGTGTTTGACCTGGGT R: TCAAAGTCTTCCCCACCCAG	118
Hspb1	1.472**	1.911***	NM_013560.2	F: ACTGGCAAGCACGAAGAAAG R: AGGGAAGAGGACACTAGGGT	110
Klf10	1.582***	1.252**	NM_001289471.1	F: GTCACATCTGTAGCCACCCA R: CCTTTCACAGCCTTTCCAGC	122
Klhl40	0.645***	0.771	NM_028202.3	F: CTACTGTGCATCCCTGTCCA R: GGTCCTCCTTGTTGTCCTCA	116
Nov	0.782**	0.602***	NM_010930.5	F: GAGATGAGACCCTGTGACCA R: TCAAACTTCTCTCCGTTGCG	143
Nr1d1	0.659***	0.875	NM_145434.4	F: TTTGCCAAACACATCCCAGG R: TGTCTGGTCCTTCACGTTGA	126
Nr4a1	2.108***	1.068	NM_010444.2	F: CTCTGGTTCCCTGGACGTTA R: CAGGAAGGCAGACTCTAGCA	103
Nr4a3	1.956***	1.087	NM_015743.3	F: CGTCTGCCTTCCAAACCAAA R: TCTCTGGGTGTTGCATCTGT	122
Nupr1	1.502**	1.454	NM_019738.1	F: CCCTTCCCAGCAACCTCTAA R: AGCTTCTCTCTTGGTCCGAC	124
Pdia4	1.591***	1.359***	NM_001368756.1	F: GTCTCTGGCTACCCGACTTT R: GCCCAGACTGCTCAATCATG	109
Per2	1.736***	1.116	NM_011066.3	F: CCGTGTCAGTGTTGGGAAAC R: CATAGCCCGAGTGTACCCTC	149
Ptgds	0.839*	1.559***	NM_008963.3	F: CCTTGCTTTGTCCACATTGC R: AATCCCAAGAGACCCAGGAG	113
Spry4	1.613***	1.196*	NM_011898.3	F: AGTAGCAGCACTTCCTCCGA R: CAGCGGCTTACAGTGAACCA	120
Sstr2	1.160	1.671***	NM_001042606.3	F: CCCATCCTGTACGCCTTCTT R: TCTCCGTGGTCTCATTCAGC	142

## Discussion

People are exposed to cranial irradiation for the treatment of many brain tumors, including primary and metastatic brain tumors and head and neck malignancies^15^. However, patients with radiotherapy are concerned about the impact of ionizing radiation because it can induce delayed cognitive and emotional dysfunction, impairing the quality of life of patients treated with radiotherapy^16^. Previous studies have provided scientific evidence of radiation-induced brain dysfunction. For example, a preclinical study demonstrated that a single dose of 10 Gy induced hippocampal-dependent behavioral dysfunctions by affecting hippocampal neurogenesis and neural plasticity-related signals^7,10^. In addition, mice showed impaired hippocampal-dependent cognitive functions at 1 month after cranial irradiation by the novel object recognition memory test; this impairment persisted up to 3 months following cranial irradiation with 10 Gy^8^. However, studies investigating the delayed effect of irradiation on anxiety-like behavior are still limited. To determine whether cranial irradiation impacts anxiety-like behavior, mice were subjected to sequential exploration tasks (the OF and EPM tests). Behavioral tests were conducted 3 months after irradiation, in accordance with previous studies in which irradiated mice showed delayed onset of neurocognitive abnormalities in the behavioral tasks. In the present study, cranial irradiation led to decreased exploration of the center of the OF apparatus or open arms of the EPM, indicative of increased anxiety-like behavior, by irradiated mice. These findings suggest that cranial irradiation modulates behavioral function in terms of anxiety in the chronic phase.

Previous studies have demonstrated that cranial irradiation induced the loss of neuronal stem cells (NSCs), which underwent long-lasting changes, including apoptosis, decreased proliferation, and altered differentiation^17,18^. Rodents showed deficits in hippocampal neurogenesis, and transplanted NSCs failed to differentiate into neurons in the irradiated brain^19^. Although numerous studies have reported the effects of cranial irradiation on a variety of cellular functions, only a few studies have focused on the delayed dysregulation of gene expression in the mouse brain. The genes *Csf1r*, *Egr1*, *Fos*, *Nr4a1*, *Nr4a3*, and *Klf10,* which were found in our study to be differentially expressed after cranial irradiation, are involved in cell differentiation. *Csf1r*, which regulates the survival, proliferation, and differentiation of microglia^20^, was downregulated following cranial irradiation, in line with the findings that Iba1, a microglial marker, was decreased in the chronic phase^10^. Moreover, a previous study reported that *Klf10* plays an important role as a tumor suppressor, and overexpression of *Klf10* downregulates cell proliferation in many cancers^21^. This study showed that *Klf10* expression was increased in the cortex and hippocampus following cranial irradiation, which might be associated with dysregulated cell differentiation^19^. Additionally, the radiation-induced disturbance of immediate-early genes (IEGs) in the hippocampus by contextual fear conditioning has been reported^6^. Our results showed that cranial irradiation induced disturbance in the expression of IEGs, including *Egr1* and *Fos*, which have been implicated in neuronal plasticity and memory formation in the brain^22^. *Nr4a1* and *Nr4a3*, nuclear receptors that function as transcription factors, have been implicated in the regulation of IEGs^23^. This is consistent with our results showing that the expression of *Nr4a1* as well as *Egr1* and *Fos* was induced following cranial irradiation.

The cellular response to radiation is complicated and involves DNA damage and the apoptosis of neuronal progenitor cells, which is partially p53-mediated^24^. *Cdkn1a*, an important effector of p53-mediated G1 arrest in response to many stresses, was found to be upregulated in the irradiated brain and regulated an increase in glioblastoma recurrence^25^. In addition, previous results have shown the upregulation of *Gadd45a* and demonstrated its potential as a biomarker for biological dosimetry in radiation therapy and early-response accidents^26^. The overexpression of *Hspb1*, which is involved in the metastasis and susceptibility of tumor cells, has been detected in patients with tumors^27^. A previous in vivo study demonstrated that knockdown of *Hspb1* enhanced the cytotoxic effects of radiotherapy^28^, indicating the involvement of *Hspb1* in the resistance of tumor cells to radiotherapy. In the present study, we found that while the mRNA expression levels of *Gadd45a* were significantly increased in the cortex, the *Cdkn1a* and *Hspb1* genes were upregulated in both the cortex and hippocampus in the chronic phase following cranial irradiation. These data demonstrated that *Cdkn1a* and *Hspb1* can be used as potential biomarkers for radiation-induced behavioral dysfunction, especially anxiety-like behavior, in the chronic phase.

Previous studies have suggested that irradiated mice display depression-like behavior^10,29^. In addition, the disturbance of circadian rhythms was reported in patients with mood disorders^30^ and anxiety disorders^31^, indicating the involvement of circadian rhythm in the pathogenesis of anxiety and mood disorders. Disruption of circadian rhythms by the injection of viral vectors induced helplessness and anxiety-like behavior in mice^32^. A previous study reported decreased levels of clock genes, including *Ciart* and *Per2*, in the brains of ketamine-treated mice^33^. In addition, the patterned expression of circadian genes, including *Per2* and *Nr1d1*, was dysregulated in the brains of patients with major depressive disorder^34^. In the present study, downregulation of clock genes, including *Ciart* and *Nr1d1,* was observed in the brains of irradiated mice, which suggested that the disruption of genes related to circadian rhythm might be related to radiation-induced behavioral dysfunction. The physiological and behavioral significance of circadian timing is complex, and further studies are required to demonstrate time- and dose-dependent changes in genes that regulate circadian rhythms by radiation exposure.

In conclusion, we provide the first evidence that cranial irradiation induces anxiety-like behavior in mice during the chronic phase, possibly via alterations in the expression of genes in the mouse cortex and hippocampus. These molecular targets, revealed by RNA-seq, might serve as biomarkers for radiation-induced behavioral dysfunctions, especially anxiety-like disorders. Cranial irradiation affects a wide range of biological processes linked to cell differentiation, circadian behavior, and kinase activity. Consequently, we suggest that anxiety-like behavior in the chronic phase may be related to alterations in the *Cdkn1a*, *Ciart*, *Fos*, *Hspa5*, *Hapb1* and *Klf10* genes, and additional analysis, including WGCNA, are required to compare RNA-seq data with behavioral dysfunctions (e.g. memory impairment, depression, and anxiety) in a variety of brain regions in irradiated mice.

## Materials and methods

### Animals

Male, 7-week-old C57BL/6 mice purchased from Orientbio, Inc. (Seongnam, Republic of Korea) were acclimatized for 1 week before the experiments were performed. All mice were housed in an SPF animal facility and had ad libitum access to tap water and commercial rodent chow. After acclimatization, the mice were randomly divided into the sham irradiation (0 Gy, *n* = 10) and irradiation (10 Gy, *n* = 10) groups. The Institutional Animal Care and Use Committee of KIRAMS approved the study protocol (KIRAMS-2021–0064), and experiments were conducted in accordance with the inter-nationally accepted principles for laboratory animal use and care dictated by the ARRIVE guidelines^35^. Every effort was made to minimize the number of animals used and their suffering.

### Irradiation

Animals received a single dose of 10 Gy irradiation using the X-RAD 320 platform (Precision X-ray, North Branford, CT) with a dose rate of 2.0 Gy/min. Mice were irradiated to the whole brain with a 20 mm × 100 mm field size. Sham-irradiated mice were placed on an identical platform for the same duration as the irradiation group but were not irradiated.

### Behavioral testing

#### Open-field (OF) test

Locomotor activity was tested by the OF test in a novel environment 90 days after cranial irradiation. Mice were individually placed into an open acrylic chamber (30 × 30 × 30 cm) for exploration. A camera was placed above the arena, and the movements of the mice were recorded for video tracking. Various parameters, including time spent in the center (central 25% of the chamber), % distance from the center, total distance, and number of entries, were analyzed with the aid of the SMART 3.0 program (Panlab, Barcelona, Spain).

#### Elevated plus maze (EPM) test

The EPM apparatus contained two open arms (25 × 5 cm) and two closed arms (25 × 5 cm), which were connected to a central platform (5 × 5 cm). The apparatus was set 50 cm above the floor. Mice were placed individually on the central platform, and they could freely explore the apparatus for 8 min. The time spent in each arm and distances traveled along each arm were analyzed by using tracking software (SMART 3.0).

### RNA extraction

Total RNA was isolated from the cortex and hippocampus using an RNeasy Lipid Tissue Mini Kit (Qiagen, Valencia, CA, USA) according to the manufacturer’s instructions. The concentration of RNA in the samples was quantified by optical densitometry using a NanoDrop ND One spectrophotometer (Thermo Fisher Scientific, Waltham, MA, USA).

### RNA sequencing (RNA-seq)

#### Library preparation and sequencing

Libraries were prepared from total RNA using the NEBNext Ultra II Directional RNA-Seq Kit (New England Biolabs, Inc., UK). mRNA was isolated using the Poly(A) RNA Selection Kit (Lexogen, Inc., Austria). The isolated mRNA was used for cDNA synthesis and shearing following the manufacturer’s instructions. Indexing was performed using Illumina indexes 1–12. The enrichment step was carried out using PCR. Subsequently, the libraries were checked using TapeStation HS D1000 screen tape (Agilent Technologies, Amstelveen, The Netherlands) to evaluate the mean fragment size. Quantification was performed using a library quantification kit and the StepOne Real-Time PCR System (Life Technologies, Inc., USA). High-throughput sequencing was performed as paired-end 100 sequencing using NovaSeq 6000 (Illumina, Inc., USA).

#### Analysis of RNA-seq data

Quality control of raw sequencing data was performed using FastQC (Simon, 2010). Reads containing adapter and low-quality reads (< Q20) were removed using FASTX_Trimmer^36^ and BBMap^37^. Then, the trimmed reads were mapped to the reference genome using TopHat^38^. The Read Count (RC) data were processed based on the FPKM + geometric normalization method using EdgeR within R^39^. Fragments per kb per million reads (FPKM) values were estimated using Cufflinks^40^. Data mining and graphic visualization were performed using ExDEGA (Ebiogen Inc., Korea).

### Quantitative real-time RT–PCR (qRT–PCR)

cDNA was prepared using random primers (Toyobo Inc., Tokyo, Japan) according to the manufacturer’s instructions and stored at -20 °C. qRT–PCR amplification was performed using PowerUP 2X SYBR Green Master Mix (Thermo Fisher Scientific) on a StepOne Real-Time PCR System (Applied Biosystems, CA, USA) according to the manufacturer’s instructions. The primer sequences are shown in Table 2. All data were normalized by reference to the amplification levels of the glyceraldehyde-3-phosphate dehydrogenase (GAPDH) gene; a reference dye was included in the SYBR Master Mix. Thresholds calculated by the software were used to calculate specific mRNA expression levels using the cycle-at-threshold (Ct) method, and all results are expressed as the fold change (compared to control) in each transcript determined employing the 2^−ΔΔ CT^ approach.

### Statistical analysis

Data are expressed as the mean ± SE. Differences between the results from the sham-irradiated and 10 Gy-irradiated groups were evaluated by two-tailed Student’s t tests using GraphPad Prism 9 software (GraphPad Software; San Diego, CA, USA). A p value less than 0.05 was considered to indicate statistical significance.

## Supplementary Information

Supplementary Information 1.

Supplementary Information 2.

## Data Availability

RNA-seq data used in this study are deposited in the Gene Expression Omnibus (GEO, https://www.ncbi.nlm.nih.gov/geo/) under the accession number of GSE204993.
