# Supplementary material for: Profiling of gene expression in the brain associated with anxiety-related behaviors in the chronic phase following cranial irradiation
Source: Sci Rep. 2022 Aug 1;12:13162. doi: 10.1038/s41598-022-17310-z (PMC9343641; doi:10.1038/s41598-022-17310-z)
Supplement: Supplementary file 1 — Supplementary Information 1. [file 41598_2022_17310_MOESM1_ESM.docx]

# Supplementary Figure legend

**Figure S1.** The clustering heatmap of 181 DEGs between sham-irradiated (Con) and irradiated (IR) mice. Each column represents the fold change in the sample (n=3/group), and each row represents the expression level of a single gene.
